# The Nutraceutical Dehydrozingerone and Its Dimer Counteract Inflammation- and Oxidative Stress-Induced Dysfunction of* In Vitro* Cultured Human Endothelial Cells: A Novel Perspective for the Prevention and Therapy of Atherosclerosis

**DOI:** 10.1155/2016/1246485

**Published:** 2016-12-05

**Authors:** Elisabetta Profumo, Brigitta Buttari, Daniela D'Arcangelo, Lavinia Tinaburri, Maria Antonietta Dettori, Davide Fabbri, Giovanna Delogu, Rachele Riganò

**Affiliations:** ^1^Department of Infectious, Parasitic and Immune-Mediated Diseases, Istituto Superiore di Sanità, Viale Regina Elena 299, 00161 Rome, Italy; ^2^Laboratory of Vascular Pathology, Istituto Dermopatico Dell'Immacolata-IRCCS, Fondazione Luigi Maria Monti, Via Monti Creta 104, 00167 Rome, Italy; ^3^Istituto di Chimica Biomolecolare, CNR, Traversa La Crucca 3, 07100 Sassari, Italy

## Abstract

Atherosclerosis is characterized by endothelial dysfunction, mainly induced by inflammation and oxidative stress. Increased reactive oxygen species (ROS) production together with increased adhesion molecules and thrombogenic tissue factor (TF) expression on endothelial cells has a key role in proatherogenic mechanisms. Therefore downmodulation of these molecules could be useful for reducing the severity of inflammation and atherosclerosis progression. Dehydrozingerone (DHZ) is a nutraceutical compound with anti-inflammatory and antioxidant activities. In this study we evaluated the ability of DHZ and its symmetric dimer to modulate hydrogen peroxide- (H_2_O_2_-) induced ROS production in human umbilical vein endothelial cells (HUVEC). We also evaluated intercellular adhesion molecule- (ICAM-) 1, vascular cell adhesion molecule- (VCAM-) 1, and TF expression in HUVEC activated by tumor necrosis factor- (TNF-) *α*. HUVEC pretreatment with DHZ and DHZ dimer reduced H_2_O_2_-induced ROS production and inhibited adhesion molecule expression and secretion. Of note, only DHZ dimer was able to reduce TF expression. DHZ effects were in part mediated by the inhibition of the nuclear factor- (NF-) *κ*B activation. Overall, our findings demonstrate that the DHZ dimer exerts a potent anti-inflammatory, antioxidant, and antithrombotic activity on endothelial cells and suggest potential usefulness of this compound to contrast the pathogenic mechanisms involved in atherosclerosis progression.

## 1. Introduction

Endothelial dysfunction is considered an initial step in the pathogenesis of atherosclerosis [1, 2]. It is mainly induced by inflammation and oxidative stress leading to structural and functional changes in the vascular endothelium. Functional changes of endothelial cells (ECs) include an increase of leukocyte adhesiveness and leukocyte diapedesis [2]. Following activation by inflammatory cytokines such as tumor necrosis factor- (TNF-) *α*, ECs express chemotactic proteins responsible for leukocyte recruitment [3]. Leukocyte infiltration into the arterial wall leads to the development of atherosclerotic lesion and it is mediated by the induction of intercellular adhesion molecule- (ICAM-) 1 and vascular cell adhesion molecule- (VCAM-) 1 on the surface of dysfunctional ECs [4, 5]. ICAM-1 and VCAM-1, membrane glycoproteins belonging to the immunoglobulin superfamily, mediate the adhesion of leukocytes to activated ECs by interacting with their ligands and induce the arrest of these cells at the vascular surface [6]. Functional changes of ECs also comprise an increased procoagulant activity [2], mediated by the induction of tissue factor (TF) on endothelial cells. TF is known to be the key element in the initiation of the coagulation cascade and appears to be a critical determinant of atherosclerotic plaque thrombogenicity [7]. TF is expressed mainly on subendothelial tissues, but its expression may be induced on endothelial cells by inflammatory mediators such as TNF-*α*. Subendothelial TF is responsible for initiating fibrin formation at sites of vascular injury whereas blood-born TF triggers atherothrombosis [7]. Due to the central role of endothelial dysfunction in atherosclerotic plaque formation and progression, the identification of novel medicinal agents able to counteract pathological functional changes of ECs is an important goal to reduce the severity of inflammation and to prevent atherosclerotic plaque development and thrombogenicity. Considering that endothelial dysfunction is implicated not only in the pathogenesis of atherosclerosis but also in a variety of pathological inflammatory conditions [3, 5, 8–13], studies aimed at identifying new anti-inflammatory molecules able to maintain endothelial homeostasis are fundamental to combat many life-threatening diseases. Previous findings indicate that some classes of antioxidants may inhibit inflammatory mediators and TF expression, in part by reducing reactive oxygen species (ROS) and targeting redox-sensitive signaling pathways that modulate inflammatory and prothrombotic processes [14].

Clinical and experimental studies indicate that many nutraceuticals, food-derived bioactive compounds, in virtue of their antioxidant and anti-inflammatory properties have the potential to reduce the risk of chronic diseases such as atherosclerosis, hypertension, and diabetes [15–19]. Among nutraceuticals, curcumin (CUR), the active component of* Curcuma longa* with anti-inflammatory, antioxidant, and anticancer properties [20–24], has been tested as a potential therapeutic agent in different pathological conditions, including cardiovascular diseases [25–30]. This natural compound has been shown to reduce the production of proatherogenic cytokines in activated human monocytes [31]. Furthermore, a randomized controlled trial has demonstrated that CUR dietary supplementation decreases cardiovascular risk in patients with type 2 diabetes [32]. However the clinical usefulness of CUR is hampered by its poor bioavailability [16] and its low solubility in aqueous solution at physiological pH. In these conditions CUR degrades, determining the production of different compounds such as vanillin, ferulic acid, and dehydrozingerone (DHZ) [33]. Although DHZ is regarded as half-analogue of CUR, it is one of the important constituents of ginger (*Zingiber officinale*), a common spice known in the world for health-promoting properties [34]. DHZ is characterized by higher solubility and stability in water [30], and, like CUR, it shows many biological activities such as a potent anti-inflammatory activity and the ability to scavenge oxygen free radicals [29, 30]. From literature data it is evident that DHZ and its derivatives can be exploited for the development of various medicinal compounds [35].

Structurally, DHZ is 2-methoxyphenol, unit present in a large class of naturally occurring compounds, to which CUR belongs. Often, dimers of 2-methoxyphenols exert higher biological activities compared to the corresponding monomer in virtue of the improved capacity to form stable radicals and to interact with a large set of proteins [36–40]. The presence of DHZ dimer in nature is likely to occur, as demonstrated for eugenol, a natural compound structurally similar to dehydrozingerone. Symmetric dimer of eugenol, namely, dehydrodieugenol, has been isolated from clove (*Eugenia caryophyllata*) and other plant extracts where it is present in mixture with its corresponding monomer [41, 42]. In a previous study the dimer of DHZ monohydrogenated at one aliphatic chain has been prepared by an enzymatic procedure using manganese (III) peroxidase (immobilized culture of* Phanerochaete chrysosporium* BKM-F-1767) [43].

We have previously demonstrated that DHZ and the corresponding symmetric dimer (DHZ dimer) protect lipids from autoxidation in combination with common antioxidants [44]. The antioxidant activity of DHZ dimer is also associated with antiaggregating and cytoprotective properties as demonstrated by its ability to partially inhibit the aggregation process of *α*-synuclein, a protein involved in neurodegenerative disorders [45].

Considering all these data, the aim of our study was to evaluate possible usefulness of DHZ and its symmetric dimer as antiatherosclerotic compounds by investigating their antioxidant, anti-inflammatory, and antithrombotic activity on human umbilical vein endothelial cells (HUVEC) exposed to oxidative and inflammatory stimuli. First of all we evaluated the ability of these compounds to affect ROS production in H_2_O_2_-stressed HUVEC. To assess their anti-inflammatory effects, we evaluated their ability to reduce the expression and secretion of the adhesion molecules ICAM-1 and VCAM-1 in HUVEC exposed to the proinflammatory cytokine TNF-*α*. To assess the antithrombotic activity of DHZ and its dimer we determined TF release by TNF-*α*-activated HUVEC. Considering that DNA binding studies have demonstrated a pivotal role of the nuclear factor- (NF-) *κ*B, the transcription factor implicated in the regulation of many immune and inflammatory responses [46], including the induction of adhesion molecule expression [47, 48] and TF expression [49–51], we evaluated the possible involvement of NF-*κ*B in the mechanisms of action of the two molecules under study.

## 2. Materials and Methods

### 2.1. Synthesis of DHZ and DHZ Dimer

DHZ and DHZ dimer were prepared according to our previous article [45].

#### 2.1.1. DHZ

To a stirred solution of vanillin (1.50 g, 9.8 mmol) in acetone (50 mL) at room temperature and under N_2_, an aqueous solution of NaOH (30 mL, 30.0 mmol) was added dropwise. The mixture was stirred at room temperature for 12 h. The solvent was evaporated under vacuum; then, water and 10% HCl were cautiously added. The organic phase was extracted with ether, dried over Na_2_SO_4_, and evaporated to afford a brown solid. The crude material was purified by flash chromatography using CH_2_Cl_2_ as eluent, to give DHZ (1.75 g, 93%): mp 127-128°C. ^1^H NMR *δ* 2.38 (s, 3H), 3.85 (s, 3H), 5.96 (bs, 1H), 6.53 (d, *J* = 16.0 Hz, 1H), 6.92 (d, *J* = 8.0 Hz, Ar, 1H), 7.04 (d, *J* = 1.6 Hz, Ar, 1H), 7.01 (dd, *J* = 1.6, 8.0 Hz, Ar, 1H), 7.42 (d, *J* = 16.0 Hz, 1H); ^13^C NMR *δ* 27.29, 56.93, 109.28, 114.81, 123.95, 124.98, 126.90, 143.77, 146.88, 148.26, 198.46.

#### 2.1.2. DHZ Dimer

To a stirred solution of dehydrodivanillin (2.00 g, 6.6 mmol) [38] in acetone (50 mL) at room temperature and under N_2_, an aqueous solution of LiOH (40 mL, 40.0 mmol) was added dropwise. The mixture was stirred at reflux for 15 h. After this period, water and 10% HCl were cautiously added. The precipitate was filtered, washed with water, and dried to afford DHZ dimer as a yellow solid (2.00 g, 80%): mp 242-243°C. ^1^H NMR *δ* 2.36 (s, 6H), 3.98 (s, 6H), 5.30 (bs, 2H), 6.60 (d, *J* = 16.0 Hz, 2H), 7.1 (d, *J* = 2.0 Hz, Ar, 2H), 7.14 (d, *J* = 2.0 Hz, Ar, 2H), 7.47 (d, *J* = 16.0 Hz, 2H); ^13^C NMR *δ* 27.32, 56.22, 108.77, 123.57, 125.27, 125.44, 126.60, 143.51, 145.45, 147.36, 198.30.

### 2.2. Cell Cultures

HUVEC (Clonetics/Lonza, Basel, Switzerland) were maintained in complete medium (EGM-2; Lonza) composed of endothelial cell basal medium (EBM-2, Lonza) supplemented with endothelial cell Bullet Kit which contains 2% foetal calf serum (FCS), human epidermal growth factor- (EGF-) 2, human fibroblast growth factor-2 (FGF-B), human vascular endothelial cell growth factor (VEGF), R3-insulin-like growth factor-1 (R3-IGF-1), ascorbic acid, hydrocortisone, heparin, gentamicin, and amphotericin B. Cells were grown at 37°C in a humidified atmosphere with 5% CO_2_/95% air, as previously described [52], and were used between passages 3 and 5. HUVEC were cultured in EBM-2 containing different concentrations of DHZ or DHZ dimer. After an overnight incubation, cells were stimulated with 10 ng/mL of human recombinant TNF-*α* (Tebu-bio, Le Perray en Yvelines, France) or treated with 300 *μ*M H_2_O_2_ (SIGMA, St. Louis, MO, USA).

The viability of HUVEC was assessed by trypan blue exclusion test. After overnight treatment with DHZ or DHZ dimer, HUVEC were harvested, stained with trypan blue 0.5% (Euroclone, Pero, MI, Italy), and counted by transmitted light microscopy using an Axioskop microscope (Carl Zeiss, Jena, Germany).

### 2.3. Intracellular ROS Production

ROS production was determined by the assessment of ROS-mediated conversion of the molecule 2′,7′-dichlorofluorescein-diacetate (DCFH-DA) into fluorescent DCFH in H_2_O_2_-treated cells. HUVEC were stained with DCFH-DA (Invitrogen Molecular Probes, Carlsbad, CA, USA) 10 *μ*M, for 30 minutes at 37°C, and then cultured in 6-well plates (Corning) (1 × 10^5^ cells/mL) in EBM-2 containing DHZ or DHZ dimer. After an overnight incubation, cells were treated with H_2_O_2_ or medium alone for 1 hour. At the end of culture, HUVEC were harvested using 0.1% Trypsin-EDTA (SIGMA) and washed and analyzed by flow cytometry using a FACSCalibur flow cytometer (Becton Dickinson) and CellQuest software.

### 2.4. ICAM-1 and VCAM-1 Surface Expression

To evaluate surface expression of ICAM-1 and VCAM-1, HUVEC (1 × 10^5^ cells/mL) were cultured in 6-well plates (Corning, Tewksbury MA, USA). After an overnight incubation with 5 *μ*g/mL or 10 *μ*g/mL DHZ or DHZ dimer, cells were stimulated with TNF-*α* or medium alone for 6 hours and harvested using 0.1% Trypsin-EDTA. HUVEC were labelled with FITC anti-human ICAM-1 antibody (BioLegend, San Diego, CA, USA) and with phycoerythrin (PE) anti-human VCAM-1 antibody (BioLegend), for 30 minutes on ice in the dark. After washing, the cells were fixed with 1% paraformaldehyde (Santa Cruz Biotechnology Inc., Santa Cruz, CA, USA). Samples were analyzed using a FACSCalibur flow cytometer (Becton Dickinson) and CellQuest software.

### 2.5. ICAM-1 and VCAM-1 Secretion

To evaluate the secretion of adhesion molecules, HUVEC were cultured as indicated for the determination of ICAM-1 and VCAM-1 surface expression. Supernatants were collected at the end of cell culture and were aliquoted at −80°C until use. Soluble ICAM-1 and VCAM-1 concentrations were quantified by the commercially available ELISA kits ICAM-1 Soluble Human ELISA Kit (Invitrogen, Frederick, MD, USA) and Human sVCAM-1 Platinum ELISA (eBioscience, San Diego, CA, USA), as recommended by the manufacturer. The limits of detection were 0.33 ng/mL for ICAM-1 and 0.6 ng/mL for VCAM-1.

### 2.6. ICAM-1 and VCAM-1 Gene Expression

To evaluate if DHZ dimer and monomer were able to modulate adhesion molecule gene expression, HUVEC (5 × 10^5^ cells/mL) were plated in 100 mm petri dish (Corning) and were cultured in EBM-2 containing 10 *μ*g/mL of DHZ or DHZ dimer. After an overnight incubation, cells were stimulated with TNF-*α* (Tebu-bio) or medium alone for 6, 24, and 48 hours.

Total cellular RNA was extracted at each time point, using the TRIzol reagent (Invitrogen- Life Technologies Italia, Monza, Italy) according to the manufacturer's instructions. First-strand cDNAs were synthesized using a mixture of 2 *μ*g of total RNA, oligo(dT)12–18 primers, and Superscript reverse transcriptase III (Invitrogen). mRNAs levels were analyzed using the SYBR-Green quantitative real-time PCR (qRT-PCR) method (5 ng per assay, Qiagen, Hilden, Germany) according to the manufacturer's instructions. Quantification was then achieved with ABI Prism 7000 SDS (Applied Biosystems, Monza, Italy). Relative expression was calculated using the comparative cycle threshold (Ct) method (2^−ΔΔCt^). mRNA expression was normalized to human ribosomal protein L13 (RPL13) levels.

The following primers for specific genes were used:

Human ICAM-1: forward 5′-ccttcctcaccgtgtactgg-3′; reverse 5′-agcgtagggtaaggttcttgc-3′
Human VCAM-1: forward 5′-tgcacagtgacttgtggacat-3′; reverse 5′-ccactcatctcgatttctgga-3′
Human RPL13: forward 5′-ggagtaccgctccaaactca-3′; reverse 5′-ggtggccagtttcagttctt-3′

### 2.7. TF Expression

To evaluate the expression of the transmembrane protein TF, HUVEC were cultured with 10 *μ*g/mL of DHZ or DHZ dimer and, after an overnight incubation, were stimulated with TNF-*α* (Tebu-bio) for 6 hours. At the end of culture cells were lysed and solubilized with 15 mM octyl-*β*-D-glucopyranoside (Abcam Inc., Cambridge, MA, USA) at 37°C for 15 minutes. TF expression was determined by a commercially available ELISA kit (Abcam Inc.), as recommended by the manufacturer. The minimum detectable dose was 4 pg/mL.

### 2.8. NF-*κ*B Translocation

To evaluate NF-*κ*B activation, HUVEC (1 × 10^5^ cells/mL) were preincubated with DHZ or DHZ dimer overnight and then treated with 10 ng/mL TNF-*α* (Tebu-bio) for 1 hour. At the end of the treatment, cells were harvested and nuclear extracts were prepared by the use of the Nuclear Extract Kit (Active Motive, Carlsbad, CA, USA). Protein content was quantified using Bradford Reagent (SIGMA), and NF-*κ*B (p65 and p50) translocation within the nucleus was assessed using NF-*κ*B p50 and NF-*κ*B p65 Transcription Factor Assay Kits (Active Motive).

### 2.9. Statistical Analysis

Results are expressed as arithmetic means and standard deviations (SD). Data were tested for Gaussian distribution with the Kolmogorov-Smirnov test and were analyzed using one-way ANOVA with a Bonferroni* post hoc* test or Student's *t*-test to evaluate the statistical significance of intergroup differences. Linear regression analysis was used to determine correlations between surface expression and soluble form concentrations of adhesion molecules. A *p* value less than 0.05 was considered statistically significant.

## 3. Results

### 3.1. Cell Viability

**Figure 1:**
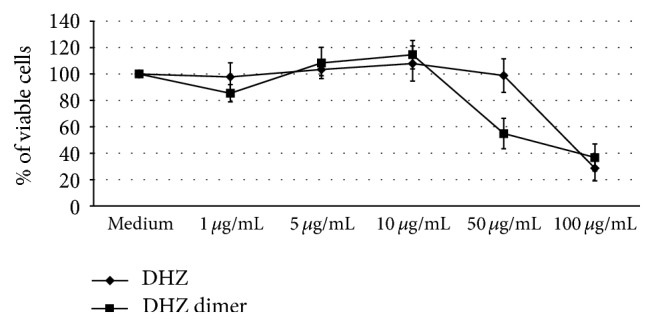
Dose-response curve of cell viability. Human umbilical vein endothelial cells (HUVEC) were exposed overnight to dehydrozingerone (DHZ) and the symmetric dimer (DHZ dimer). Cell viability was evaluated by trypan blue exclusion analysis (mean ± SD of three independent experiments).

Preliminary dose-response experiments conducted to evaluate a possible toxic effect of DHZ dimer and monomer on ECs showed a reduction of cell viability after a treatment with 50 *μ*g/mL of DHZ dimer and with 100 *μ*g/mL of monomer (Figure 1). The highest tolerated concentrations (5 and 10 *μ*g/mL) that did not affect cell viability were used to investigate the antioxidant, anti-inflammatory, and antithrombotic activity of DHZ compounds on ECs.

### 3.2. Intracellular ROS Production

**Figure 2:**
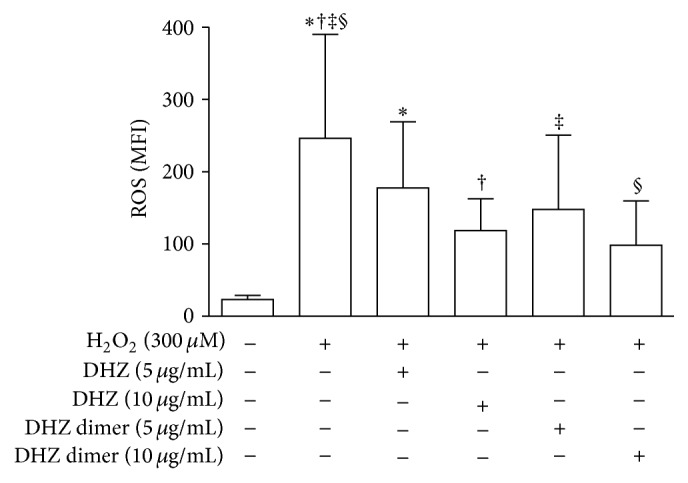
Cytofluorimetric analysis of intracellular reactive oxygen species (ROS) production. ROS production is expressed as mean fluorescence intensity (MFI) in HUVEC pretreated with DHZ or DHZ dimer and then treated with hydrogen peroxide (H_2_O_2_) 300 *μ*M for 1 hour (mean values of four experiments). Error bars represent SD. ^*∗*^ *p* = 0.0347; ^†^ *p* = 0.0477; ^‡^ *p* = 0.0113; ^§^ *p* = 0.0138. Medium versus H_2_O_2_, *p* > 0.01.

To evaluate the antioxidant activity of DHZ and DHZ dimer on ECs we assessed the ability of these compounds to counteract ROS production induced by H_2_O_2_ treatment. Cytofluorimetric analysis showed that the two compounds, at both the concentrations used, were able to reduce intracellular ROS production expressed as mean fluorescence intensity (MFI) on HUVEC exposed to 300 *μ*M of H_2_O_2_ (Figure 2). The comparison of the two compounds showed a higher antioxidant activity of the DHZ dimer, even if the differences observed were not statistically significant.

### 3.3. Surface Expression of ICAM-1 and VCAM-1

**Figure 3:**
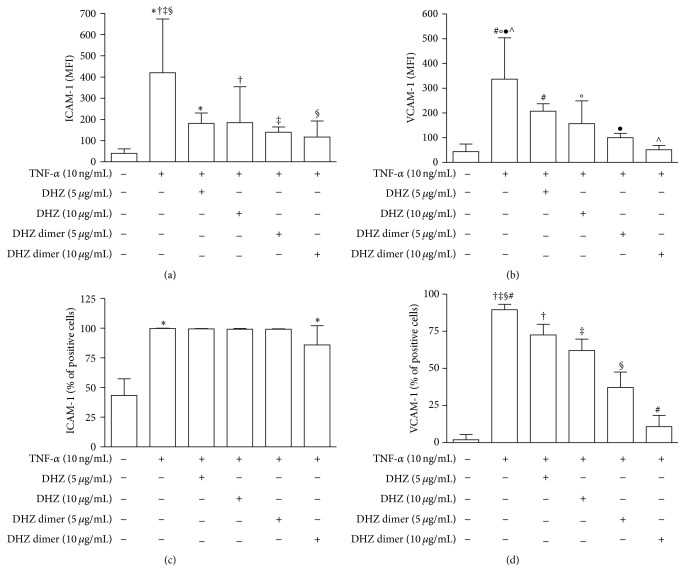
Cytofluorimetric analysis of adhesion molecule surface expression. HUVEC were pretreated with DHZ or DHZ dimer and then treated with TNF-*α* 10 ng/mL for 6 hours. (a) and (b) show ICAM-1 and VCAM-1 expression evaluated as mean fluorescence intensity (MFI) (mean values of six experiments); (c) and (d) show ICAM-1 and VCAM-1 expression evaluated as percentages of positive cells (mean values of six experiments). Error bars represent SD. (a) ^*∗*^ *p* = 0.0242; ^†^ *p* = 0.0350; ^‡^ *p* = 0.0061; ^§^ *p* = 0.0047. Medium versus TNF-*α*, *p* < 0.01. (b) ^#^ *p* = 0.0242; °*p* = 0.0221; ^●^ *p* = 0.0061; ^∧^ *p* = 0.0012. Medium versus TNF-*α*, *p* < 0.001. (c) ^*∗*^ *p* = 0.0061. Medium versus TNF-*α*, *p* < 0.001. (d) ^‡, #^ *p* = 0.0012; ^†, §^ *p* = 0.0061. Medium versus TNF-*α*, *p* < 0.001.

**Figure 4:**
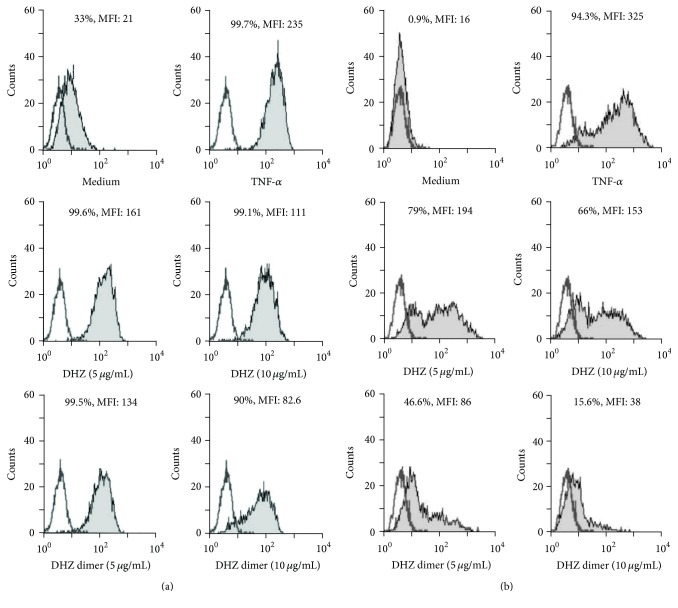
Adhesion molecule surface expression. Representative histograms showing cytofluorimetric analysis of ICAM-1 (a) and VCAM-1 (b) surface expression. Empty histograms represent isotype controls.

Cytofluorimetric analysis of adhesion molecule surface expression showed a significant reduction of TNF-*α*-induced ICAM-1 and VCAM-1 surface expression evaluated as MFI in response to both DHZ and DHZ dimer (Figures 3(a) and 3(b)). When adhesion molecule expression was evaluated in terms of percentage of positive cells, we observed that the two compounds were able to significantly prevent upregulation of VCAM-1 expression, whereas only the DHZ dimer was able to prevent ICAM-1 expression (Figures 3(c) and 3(d)). The comparison of the two compounds showed that the DHZ dimer was more effective than DHZ. Figures 4(a) and 4(b) show representative histograms of ICAM-1 and VCAM-1 expression observed in HUVEC preincubated with DHZ and DHZ dimer and then treated with TNF-*α*.

### 3.4. Soluble ICAM-1 and VCAM-1 Concentrations

**Figure 5:**
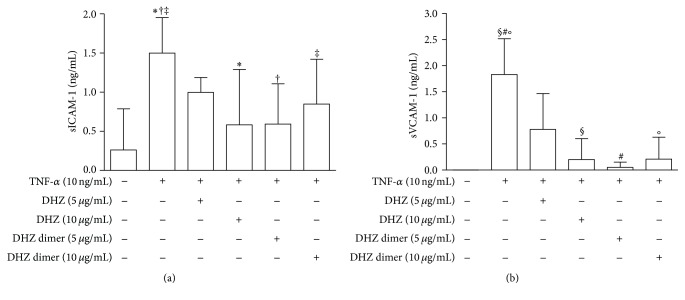
ICAM-1 and VCAM-1 secretion. Analysis of soluble ICAM-1 (a) and VCAM-1 (b) concentrations (ng/mL) by ELISA assay performed in culture supernatants from HUVEC pretreated with DHZ or DHZ dimer and then treated with TNF-*α* 10 ng/mL for 6 hours (mean values of four experiments). Error bars represent SD. (a) ^*∗*, †^ *p* = 0.0317; ^‡^ *p* = 0.0236. Medium versus TNF-*α*, *p* < 0.01. (b) ^§, #, °^ *p* = 0.0079. Medium versus TNF-*α* *p* < 0.001.

Analysis of ICAM-1 and VCAM-1 concentrations in culture supernatants from TNF-*α*-stimulated HUVEC showed lower levels of these molecules in samples pretreated with DHZ and DHZ dimer compared to those from non-pretreated samples (Figures 5(a) and 5(b)). No significant differences were observed between the two compounds. Of note, significant positive correlations were observed between ICAM-1 and VCAM-1 surface expression (MFI) and soluble form concentrations (pg/mL) in culture supernatants (ICAM-1: *r* = 0.743, *p* < 0.0001; VCAM-1: *r* = 0.769, *p* < 0.0001) (data not shown).

### 3.5. ICAM-1 and VCAM-1 Gene Expression

**Figure 6:**
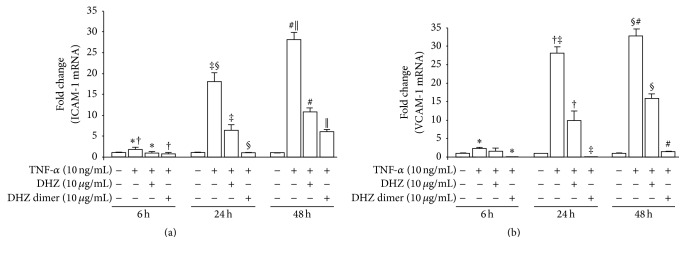
ICAM-1 and VCAM-1 gene expression. ICAM-1 (a) and VCAM-1 (b) expression analysis by quantitative real-time PCR (qRT-PCR). HUVEC were cultured in endothelial cell basal medium containing 10 *μ*g/mL of DHZ or DHZ dimer or medium alone. After an overnight incubation, cells were stimulated with 10 ng/mL of human recombinant TNF-*α* or medium alone for 6, 24, and 48 hours. Total cellular RNA was extracted at each time point and mRNA levels were analyzed. Error bars represent SD. (a) ^*∗*, #^ *p* = 0.004; ^†^ *p* = 0.003; ^‡^ *p* = 0.001; ^§^ *p* = 0.0002; ^||^ *p* = 0.002. (b) ^*∗*^ *p* = 0.004; ^†^ *p* = 0.0004; ^‡^ *p* = 0.0006; ^§^ *p* = 0.0002; ^#^ *p* = 0.0005.

Gene expression analysis of adhesion molecules by qRT-PCR at 6, 24, and 48 hours of incubation showed a significant increased expression of both ICAM-1 and VCAM-1 at each time point, in TNF-*α* -treated cells. In all cases, preincubation with DHZ and DHZ dimer significantly reduced such expression (Figures 6(a) and 6(b)), confirming their potential anti-inflammatory activity. Again, the comparison of the two compounds showed that the DHZ dimer was more effective than DHZ.

### 3.6. TF Expression

**Figure 7:**
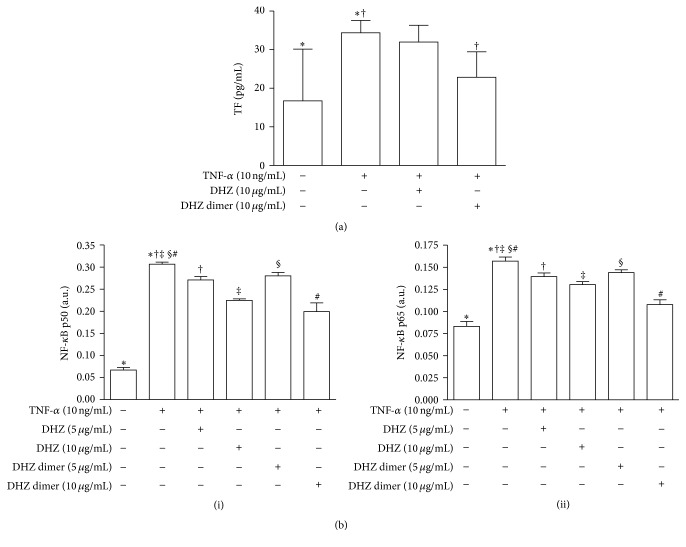
Tissue factor (TF) expression and nuclear factor-*κ*B activation. (a) shows the detection of TF expression on cell membrane in HUVEC pretreated with DHZ or DHZ dimer and then treated with TNF-*α* 10 ng/mL for 6 hours (mean values of four experiments). (b) shows the evaluation of p50 (i) and p65 (ii) translocation within the nucleus in HUVEC pretreated with DHZ or DHZ dimer and then treated with TNF-*α* 10 ng/mL for 1 hour (mean values of four experiments). Error bars represent SD. (a) ^*∗*^ *p* = 0.043; ^†^ *p* = 0.02. (b) (i) and (ii) ^*∗*, †, ‡, #^ *p* < 0.001; ^§^ *p* < 0.05; a.u.: arbitrary units.

To assess the possible antithrombotic activity of DHZ and DHZ dimer, we evaluated TF expression on cell membrane. TNF-*α* treatment significantly increased TF expression in HUVEC compared to medium alone (Figure 7(a)). DHZ dimer but not DHZ was able to downmodulate TF expression (Figure 7(a)).

### 3.7. NF-*κ*B Activation

To evaluate NF-*κ*B activation we assessed p50 and p65 translocation within the nucleus. The exposure to TNF-*α* significantly increased active NF-*κ*B p50 and p65 levels in HUVEC when compared to untreated cells (Figure 7(b), (i) and (ii)). When HUVEC were pretreated with DHZ or DHZ dimer a significant reduction of active p50 and p65 levels was observed (Figure 7(b), (i) and (ii)).

## 4. Discussion

Our data here demonstrate that DHZ and its dimer exert antioxidant and anti-inflammatory activities on human endothelial cells, thus suggesting a possible usefulness of these compounds in the prevention and therapy of atherosclerosis. The DHZ dimer exerts a more potent activity than the monomer. Dimer is more able than DHZ to prevent H_2_O_2_-induced ROS production by ECs thus protecting these cells from oxidative stress. It is also more efficacious than its monomer in preventing TNF-*α*-induced upregulation of ICAM-1 and VCAM-1 on EC surface and their secretion in culture supernatants. Of note, DHZ dimer but not DHZ is able to inhibit TF expression on ECs. When we consider a possible intracellular signaling pathway involved in the beneficial effects of the two compounds, we found that activation of NF-*κ*B, the transcription factor implicated in the regulation of immune and inflammatory responses [46], including ICAM-1 and VCAM-1 expression [48], is downmodulated by DHZ and its dimer. To the best of our knowledge, in this study we provide the first evidence of the biological effects of DHZ and its dimer on human ECs.

Endothelial cell damage is a key event in atherosclerotic disease onset and progression [53, 54]. In physiological conditions, vascular endothelium finely modulates vascular tone and accomplishes anticoagulant, antiplatelet, and fibrinolytic activities. The vascular tone is maintained by the release of different dilator and constrictor factors. Injury of ECs hampers the balance between vasoconstriction and vasodilation and promotes prooxidant, proinflammatory, and procoagulant mechanisms that favor the development of atherosclerosis [53]. The main vasodilator factor is nitric oxide (NO) that is synthesized by the enzyme endothelial nitric oxide synthase (eNOS) and induces vascular smooth muscle relaxation. A previous study by Smith et al. performed in aged rats demonstrated that the age-related decline of endothelial functions, which directly contributes to progression of cardiovascular disease, may be partly due to the reduction of eNOS activity, with consequent decline of NO bioavailability [55]. In another study the same authors reported an age-related decline of the endothelial glutathione levels, an increase of circulating TNF-*α* concentrations, and an altered eNOS phosphorylation pattern [56]. They speculated that a prooxidant and proinflammatory environment in the aging vessel may dysregulate eNOS activity and NO bioavailability, thus promoting endothelial dysfunction.

Molecules with systemic antioxidant, anti-inflammatory, and antithrombotic activities may be useful for the prevention of atherosclerotic disease. Experimental and clinical studies have demonstrated that several nutraceuticals have antiatherogenic effects being able to contrast the biological mechanisms responsible for the formation of the atherosclerotic lesions. These compounds are of interest for the development of new therapeutic approaches because usually they have a good tolerability and less adverse side effects [57]. Our data showing that pretreatment with DHZ and DHZ dimer reduces ROS levels in ECs exposed to oxidative stress are in accordance with previous results demonstrating that CUR improves cell viability and reduces ROS production in HUVEC treated with H_2_O_2_ [30]. Our results are also in line with studies demonstrating that DHZ and its analogs hold a potent ability to scavenge oxygen free radicals [45, 58, 59]. In an* in vitro* cell model with vascular smooth muscle cells, the antioxidant activity of DHZ was at least in part responsible for its inhibitory effect on the activation and function of this cells, a key step occurring during atherosclerotic lesion progression [59]. In another study, experiments in cell free systems have demonstrated that DHZ was able to scavenge superoxide and nitric oxide radicals and to reduce Fe (III) to Fe (II) [58]. In the same study, by the use of animal models exposed to whole body gamma irradiation, it has been observed that the pretreatment with DHZ significantly increased the endogenous antioxidant enzymes and reduced the radiation-induced mortality, thus demonstrating a relevant radioprotective activity of DHZ associated to its antioxidant properties.

A prooxidant microenvironment and proinflammatory mediators, including TNF-*α*, activate ECs and upregulate the expression of adhesion molecules such as ICAM-1 and VCAM-1 on endothelium [3, 60, 61]. These molecules support the interaction of leukocytes with ECs, thus promoting their migration through the endothelial layer into the intima [62–64]. Apart from being expressed on the surface of ECs, adhesion molecules are also secreted in the extracellular compartment. Soluble forms of vascular adhesion molecules retaining their functional characteristics have been detected in the blood of healthy subjects, and increased levels have been observed in patients with pathologies characterized by vascular events [65, 66]. In a prospective cohort study designed to evaluate markers of coronary risk, increased plasma levels of ICAM-1 were associated with the risk of myocardial infarction and angina pectoris, indicating circulating levels of ICAM-1 as possible risk markers for future coronary events [67].

The potent inhibitory effects of DHZ dimer and at less extent of its monomer on adhesion molecule expression and secretion by ECs strongly suggest that these compounds could be useful in hampering leukocyte recruitment and inflammation, thus contrasting atherosclerotic disease progression.

In physiological conditions, ECs present an anticoagulant phenotype.* In vitro* studies have demonstrated that, in the presence of proinflammatory cytokines such as TNF-*α*, endothelial cells change their phenotype in a procoagulant one characterized by TF expression [68]. TF expression by endothelium has been associated with the occurrence of thrombotic events in patients with a variety of clinical disorders including atherosclerosis [68]. The proinflammatory microenvironment characteristic of human atherosclerotic plaque induces the production of TF by various cell types including ECs, vascular smooth muscle cells, and monocytes [69]. If the atherosclerotic lesion undergoes rupture, TF is released into the bloodstream [70, 71] and activates the coagulation pathway, thus promoting thrombotic events [72].

Our data showing that pretreatment of HUVEC with DHZ dimer before stimulation with TNF-*α* is able to prevent TF expression suggest that this compound exerts an anticoagulant activity and may offer therapeutic perspectives to prevent thrombus formation and acute events in patients with atherosclerotic disease.

Our findings on the reduction of p50 and p65 translocation into the nucleus observed when HUVEC were pretreated with DHZ compounds before stimulation with TNF-*α* indicate that these two compounds exert their biological effect on ECs activation at least in part via the regulation of NF-*κ*B activation. In fact in resting conditions NF-*κ*B is present within the cytoplasm in an inactive form and translocates into the nucleus in response to different stimuli such as infection, inflammation, and oxidative stress [73]. These data are in line with previous results demonstrating that zingerone, an analogous of DHZ characterized by the absence of a conjugated double bond, was able to suppress oxidative stress and age-related inflammation in kidney and endothelial cells from rats through the modulation of mitogen-activated protein kinase pathway and the inhibition of NF-*κ*B signaling [74].

Our findings here showing that DHZ dimer is more active than DHZ in contrasting EC dysfunction are in accordance with our recent results demonstrating that the dimer of DHZ is more effective than DHZ in protecting lipids from autoxidation [44] and in controlling misfolding of *α*-synuclein in an* in vitro* assay [45]. These results can be ascribed to the differences in the chemical structure between the two molecules and in their lipophilicity. It can be hypothesized that the DHZ dimer, a biphenyl structure, has a higher ability to pass the cell membrane and, in virtue of the biphenylic hydroxylated structure, it would interact with cell components more efficiently than the corresponding monomer [37].

## 5. Conclusions

Overall, our results here add new significant information and further support to previous findings on the usefulness of the nutraceutical DHZ and its analogs, which are degradants of CUR with less side effects and improved bioavailability. In particular, the symmetric dimer of DHZ, through its potent inhibitory effects on adhesion molecule and TF expression and on ROS production in endothelial cells, could be effective in contrasting the proinflammatory, prooxidant, and prothrombotic harmful mechanisms against endothelial cells thus representing a promising new medicinal compound to develop new dietary strategies for the prevention and treatment of atherosclerosis and its acute events and of other inflammatory diseases where endothelial dysfunction is a key pathogenic factor. However, we have to take into account that our results have been obtained in an* in vitro* biological system and that* in vivo* studies are necessary to confirm the effects of DHZ and its dimer and to test the concentrations that are effective* in vivo*, also considering that natural compounds are in general immediately metabolized upon ingestion.

In conclusion, in the present work we extended previous results on DHZ and its dimer and highlighted for the first time a potent effect of DHZ dimer to counteract human endothelial cell dysfunction. In virtue of the chemical stability and low toxicity of this compound for human cells [45] it exhibits potential usefulness in the prevention and therapy of several inflammatory diseases such as atherosclerosis.
